# Lactate Fluxes and Plasticity of Adipose Tissues: A Redox Perspective

**DOI:** 10.3389/fphys.2021.689747

**Published:** 2021-06-30

**Authors:** Damien Lagarde, Yannick Jeanson, Jean-Charles Portais, Anne Galinier, Isabelle Ader, Louis Casteilla, Audrey Carrière

**Affiliations:** ^1^Goodman Cancer Research Center, McGill University, Montreal, QC, Canada; ^2^Department of Biochemistry, McGill University, Montreal, QC, Canada; ^3^Institut RESTORE, UMR 1301 INSERM, 5070 CNRS, Université Paul Sabatier, Toulouse, France; ^4^MetaboHUB-MetaToul, National Infrastructure of Metabolomics and Fluxomics, Toulouse, France; ^5^Institut Fédératif de Biologie, CHU Purpan, Toulouse, France

**Keywords:** lactate, redox metabolism, white adipocytes, beige adipocytes, brown adipocytes, adipose tissues, metabolic dialogs

## Abstract

Lactate, a metabolite produced when the glycolytic flux exceeds mitochondrial oxidative capacities, is now viewed as a critical regulator of metabolism by acting as both a carbon and electron carrier and a signaling molecule between cells and tissues. In recent years, increasing evidence report its key role in white, beige, and brown adipose tissue biology, and highlights new mechanisms by which lactate participates in the maintenance of whole-body energy homeostasis. Lactate displays a wide range of biological effects in adipose cells not only through its binding to the membrane receptor but also through its transport and the subsequent effect on intracellular metabolism notably on redox balance. This study explores how lactate regulates adipocyte metabolism and plasticity by balancing intracellular redox state and by regulating specific signaling pathways. We also emphasized the contribution of adipose tissues to the regulation of systemic lactate metabolism, their roles in redox homeostasis, and related putative physiopathological repercussions associated with their decline in metabolic diseases and aging.

## Introduction

**Figure 1:**
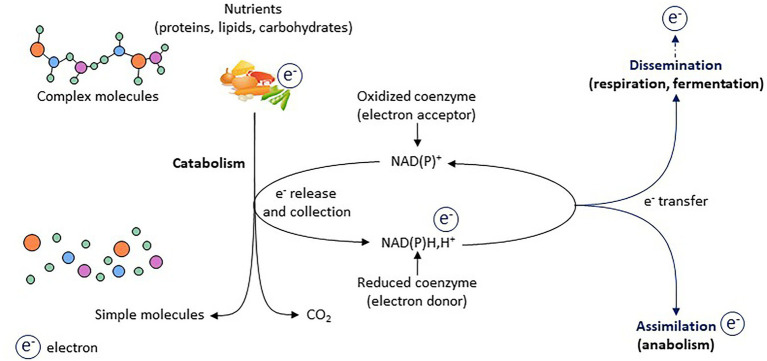
Electrons as primary source of energy for cellular activity. Electrons represent the primary source of energy in cells. During catabolic processes, electrons are released from nutrients (which are oxidized) and are accepted by co-enzymes (which become reduced). Two processes are key for regenerating oxidized forms of co-enzymes and thus for redox homeostasis: the assimilation and dissemination processes.

Energy metabolism is based on redox (i.e., reduction and oxidation reactions) metabolism that corresponds to a finely tuned network of electron transfer between molecules. Oxidative catabolism releases electrons, which are accepted by coenzymes such as NADP^+^ and NAD^+^, which then become reduced (i.e., NADPH,H^+^ and NADH,H^+^; Wu et al., 2016a; Hosios and Vander Heiden, 2018; Xiao et al., 2018). Regenerating oxidized forms of coenzymes is critical to ensure proper metabolic activity (Figure 1). Electron assimilation, which corresponds to the storage of electrons within newly synthetized molecules (anabolism), and electron dissemination, which corresponds to the release of electrons within the environment, mainly through mitochondrial respiration and lactic acid fermentation, are keys for regenerating oxidized forms of coenzymes and maintaining redox homeostasis (Figure 1). In addition to supporting energy homeostasis, the redox state drives a variety of cellular functions, such as cell proliferation, differentiation, senescence, and secretory activity. The maintenance and adjustment of redox homeostasis are essential for normal cell and tissue function, and increasing evidence demonstrates that the redox state is the primary conductor regulating metabolic adaptation during stress (Gaude et al., 2018). However, redox metabolism is inevitably associated with electron leaks and reactive oxygen species (ROS) production, which, if not properly managed, can lead to oxidative stress and its related toxicities (Holmstrom and Finkel, 2014).

**Figure 2:**
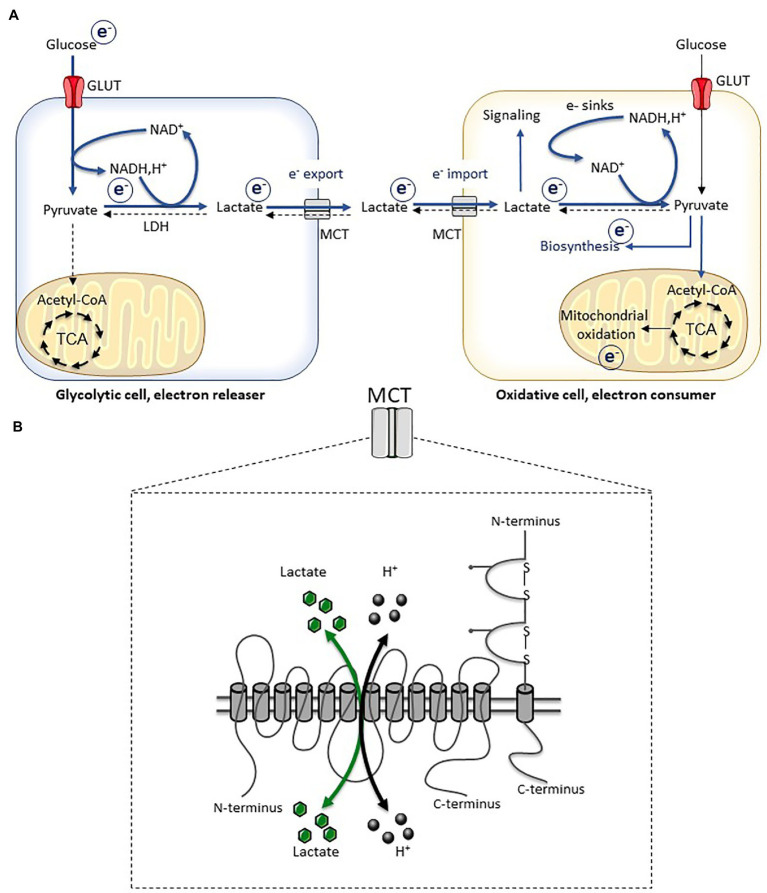
Lactate transport and redox balance. **(A)** Inter-cellular lactate exchange is associated with inter-cellular electron fluxes. **(B)** Schematic representation of monocarboxylate transporters (MCT) which are constituted by 12 transmembrane domains, and their chaperone proteins (basigin and embigin). The transport of monocarboxylates (lactate, pyruvate, and ketone bodies) is associated with a proton transport (symport) and occurs bidirectionally depending on the electrochemical gradient. Adapted from Carriere et al. (2020). TCA, tricarboxylic acid cycle.

A massive cellular network of redox enzymes, couples, thiols, and metabolites supports electron flow between molecules that cooperate to maintain redox homeostasis. Among the different factors that can communicate metabolic and redox states between cells and organs, lactate plays a key role (Brooks, 2018; Ferguson et al., 2018; Rabinowitz and Enerback, 2020). In fact, the single and reversible reaction catalyzed by lactate dehydrogenase, which is submitted to the law of mass action and depends on the relative concentrations of lactate and pyruvate, directly regulates the cytosolic NAD^+^/NADH ratio. As this redox reaction is close to equilibrium, the lactate/pyruvate ratio is considered as a marker of the cytosolic redox potential. Lactate production – which is mainly derived from glucose catabolism, although a small part can be obtained from alanine metabolism – and its export ensure redox homeostasis when the energy load overwhelms the oxidative capacities (Figure 2). Far from being a metabolic waste product, exported lactate can be consumed and oxidized either by neighboring cells or by organs at distance, making the link between glycolytic and oxidative pathways, and ensuring tissue and whole-body redox homeostasis (Brooks, 2018; Ferguson et al., 2018; Rabinowitz and Enerback, 2020).

Interorgan communication is key for maintaining whole-body energy and redox homeostasis on stresses and metabolic challenges (Castillo-Armengol et al., 2019). Among the different organs supporting such dialogs, the different types of adipose tissues (i.e., white, brown, and beige) play a key role (Cinti, 2012; Chouchani and Kajimura, 2019). In this study, we described how lactate can act as a redox signaling metabolite driving the fate of adipose cells and that lactate fluxes within adipose tissues may be key for whole-body redox homeostasis. Before exploring these aspects, we briefly described the main characteristics of lactate metabolism and adipose tissues.

## Lactate Metabolism As a Key Metabolic Pathway For the Maintenance of Redox Homeostasis

For many years, lactate has been viewed as a metabolic waste product, produced by glycolytic cells devoid of mitochondria or when glycolytic flux exceeds the oxidative capacities of cells. This historical view also associated lactate production with a potentially harmful effect, given the link between lactate and proton export, which can indeed contribute to microenvironment/blood acidification. However, lactate production is key for redox homeostasis and enables redox pressure disposal when the energy load overwhelms the oxidative capacities of a given cell (Figure 2), thereby limiting electron leaks and related oxidative stress. Once exported, lactate acts as a metabolic carrier ensuring the exchange of carbons and electrons between cells and organs. Intercellular and interorgan flows of lactate exist, and several collaborative lactate-dependent metabolic dialogs have been described as key for maintaining energy and redox homeostasis (Sonveaux et al., 2008; Pellerin and Magistretti, 2012; Rodriguez-Colman et al., 2017; Leveillard et al., 2019; Zhang et al., 2020).

### From the Cell-Centric View…

In a glycolytic cell, lactate production from pyruvate and its export out of the cell are critical for intracellular redox homeostasis, in particular, when the glycolytic flux exceeds the mitochondrial oxidative capacities. The reduction of pyruvate into lactate by the lactate dehydrogenase activity is associated with the oxidation of NADH,H^+^ into NAD^+^. This is critical for the activity of the glyceraldehyde dehydrogenase, which requires NAD^+^ to catalyze a key step of the glycolytic pathway. Since the reaction catalyzed by lactate dehydrogenase activity is reversible and is submitted to the law of mass action, export of lactate is required to enable the reaction to further moving toward the lactate production. The transport of lactate is classically described as mediated by monocarboxylate transporters (MCT), especially the isoforms 1–4 that are considered as the main lactate (also pyruvate and ketone bodies) transporters (Halestrap, 2013; Perez-Escuredo et al., 2016). The transport of lactate is associated with the transport of proton and it is bidirectional depending on the electrochemical gradient. The export of lactate through MCT, which massively occurs in a glycolytic cell, is therefore systematically associated with a simultaneous export of protons. Thus, the export of lactate does not solely correspond to an export of carbon and energy but rather to a crucial metabolic pathway for the maintenance of redox homeostasis in glycolytic cells (Figure 2). Besides the canonical MCT-dependent transport, alternative modes of lactate transport have recently been identified such as the SLC5A12 transporters in T lymphocytes (Pucino et al., 2019) or the connexin 43 in pancreatic cells (Dovmark et al., 2017).

### …To the Ecosystem-Centric View

At the cellular level, lactate production is key to manage redox pressure. At the tissue or whole organism level, the cells able to consume lactate are essential to handle electron fluxes carried out by this metabolite and to maintain redox homeostasis. In fact, if lactate can appear as a metabolic waste for some cells, it is also a substrate for many other cells throughout the body. The quantification of metabolic fluxes using *in vivo* ^13^C metabolite infusion experiments identified lactate as the main source of carbons fueling the Krebs cycle, in both physiological and pathological conditions (Faubert et al., 2017; Hui et al., 2017, 2020). Lactate also feeds biosynthesis pathways such as gluconeogenesis (Cori and Cori, 1929; Cori, 1981) and lipogenesis (Katz and Wals, 1974; Chen et al., 2016). The importance of lactate as a nutrient precursor has been demonstrated for almost 100 years when Carl Ferdinand and Gerty Theresa Cori demonstrated the role of muscle-produced lactate in hepatic gluconeogenesis (Cori and Cori, 1929; Cori, 1981). Whatever the metabolic pathways, when lactate is consumed by cells, this is associated with the import of electrons (Figure 2). In fact, lactate is oxidized into pyruvate by the lactate dehydrogenase activity and this is associated with the reduction of NAD^+^ into NADH,H^+^. Lactate-consuming cells must be able to manage these electron fluxes, through electron assimilation or dissemination processes. Mitochondrial activity corresponds to an electron dissemination process (Figure 1). If the electron load is in adequacy with the oxidative capacity of mitochondria, the production of mitochondrial ROS is low. However, if electron load overwhelms the oxidative capacities of mitochondria, the escape of electrons from the respiratory chain and the production of ROS are increased. Therefore, the rate of the lactate-dependent production of mitochondrial ROS results from the unbalance between the yield of electrons provided to the respiratory chain and the oxidative capacities, these latter depending on the quantity of mitochondria, the state of coupling/uncoupling, and the bioenergetics needs of the cells. Very interestingly, lactate by itself can activate specific signaling pathways to facilitate these electron management processes such as triggering mitochondrial biogenesis in muscle cells (Hashimoto et al., 2007) and inducing the expression of the uncoupling protein 1 (UCP1) in adipocytes (see the “Reciprocal relationships between lactate metabolism and adipose tissue biology” section; Carriere et al., 2014). These two cellular responses converge toward an increased oxidative potential, which will promote NADH,H^+^ oxidation and NAD^+^ regeneration.

## The Main Roles of Adipose Tissues in Whole-Body Energy and Redox Homeostasis

Among the different organs that cooperate to maintain whole-body energy homeostasis (Castillo-Armengol et al., 2019), adipose tissues are key not only because of their capacity to store the excess of energy under the form of triglycerides in white adipose tissues but also through the capacity of brown and beige adipose tissues to dissipate energy as heat (Chouchani and Kajimura, 2019). Lactate and redox metabolism play a critical role in the properties of each of these adipose depots (Carriere et al., 2020). Before highlighting the underlying mechanisms (see the “Reciprocal relationships between lactate metabolism and adipose tissue biology” section), following is a short description of their main characteristics.

### White Adipocytes, From Energy Storage to Energy Release

White adipocytes are specialized cells with a unique ability to store energy into triglycerides during caloric intake and to release it in the form of fatty acids during periods of caloric deficit. Following chronic caloric excess, the expansion of white adipose tissue through the increase of adipocytes size (hypertrophy) and the formation of new adipocytes from progenitors (hyperplasia) contribute to the storage of energy in excess. At the opposite, when energy has to be made available for cellular activity, white adipocytes can mobilize their lipid stores through lipolysis to release energy (Lafontan and Langin, 2009).

### Brown Adipocytes, Oxidation of Substrates, and Nonshivering Thermogenesis

Since its rediscovery in human adults in 2009 (Cypess et al., 2009; van Marken Lichtenbelt et al., 2009; Virtanen et al., 2009), the contribution of brown adipose tissue to energy expenditure and metabolic health has been intensely studied (Sidossis and Kajimura, 2015; Betz and Enerback, 2018; Hussain et al., 2020). Brown adipocytes display significant oxidative capacities due to a large number of mitochondria and the presence of the mitochondrial UCP1 located in the mitochondrial inner membrane, which uncouples the oxidation processes from ATP synthesis (Cannon and Nedergaard, 2004). Cold exposure increases the expression of UCP1 and activates its uncoupling activity, in particular, by the fatty acids released during the sympathetic activation of lipolysis (Bertholet and Kirichok, 2017). Then, energy is dissipated as heat, at the expense of ATP synthesis (Nicholls and Locke, 1984).

### Beige Adipocytes, Inducible on Stress in White Adipose Depots

Beige adipocytes display metabolic characteristics very similar to those of brown adipocytes, including UCP1 expression. However, they appear in specific regions of some white adipose tissues (Barreau et al., 2016; Dichamp et al., 2019) on metabolic challenges such as cold exposure (Young et al., 1984; Loncar, 1991; Cousin et al., 1992) through distinct cellular mechanisms (Barbatelli et al., 2010; Lee et al., 2012; Wang et al., 2013; Park et al., 2021), and the brown-like phenotype disappears once the stress is over (Rosenwald et al., 2013). Notably, several UCP1-independent thermogenic mechanisms within beige (and brown) adipocytes have been recently uncovered (Roesler and Kazak, 2020). Although cold exposure is an important driver for beiging of adipose tissue, several other stimuli have been recently highlighted such as physical exercise (Bostrom et al., 2012; Dewal and Stanford, 2019), cancer-associated cachexia (Kir et al., 2014; Petruzzelli et al., 2014; Han et al., 2018), massive burn (Porter et al., 2015; Sidossis et al., 2015), and intermittent fasting (Li et al., 2017), but the role of beige adipocytes in these contexts remained to be clarified.

### Adipose Tissues and Energy Homeostasis: A Redox View

One of the main characteristics of adipose tissues is their great plasticity, i.e., their ability to adapt their phenotype, metabolic activity, and function according to the nutritional status of the host and in response to metabolic challenges to ensure energy homeostasis (Chouchani and Kajimura, 2019). Besides this canonical view, one can consider adipose tissues as key regulators of electron fluxes, given their capacity to store electrons within triglycerides and to disseminate them, in white and brown/beige adipose tissues, respectively. In fact, in addition to carbon storage, the capacity of white adipocytes to store triglycerides can be viewed as a very efficient process of electron assimilation and storage, consistently with the reduced state observed during adipogenesis (Galinier et al., 2006). Since the high oxidation of NADH,H^+^ into NAD^+^ occurs in the presence of activated UCP1, brown and beige adipocytes may also play a key role in the redox balance by acting as controllable electron dissemination systems. These intimate relationships between adipose tissues and redox metabolism are strengthened by the growing evidence that lactate metabolism can fine-tune adipose tissue biology according to the metabolic conditions and that, reciprocally, adipose tissues impact systemic lactate metabolism.

## Reciprocal Relationships Between Lactate Metabolism and Adipose Tissue Biology

In addition to the well-known impact of adipose tissues on glucose and lipid systemic homeostasis, recent findings highlight them as regulators of other circulating metabolites, such as succinate (Mills et al., 2018, 2021) or branched-chain amino acids (Yoneshiro et al., 2019). Several metabolites have also been shown to promote adipocyte plasticity toward a beige phenotype (Sahuri-Arisoylu et al., 2016; Liu et al., 2020). In the following section, we focused on the relationships between lactate metabolism and metabolic activity of adipose tissues, highlighting that lactate can act as a signaling molecule in adipose tissues and that, reciprocally, adipose tissues can impact systemic lactate metabolism.

### Lactate Regulates the Metabolic Activity of White, Brown, and Beige Adipocytes

**Figure 3:**
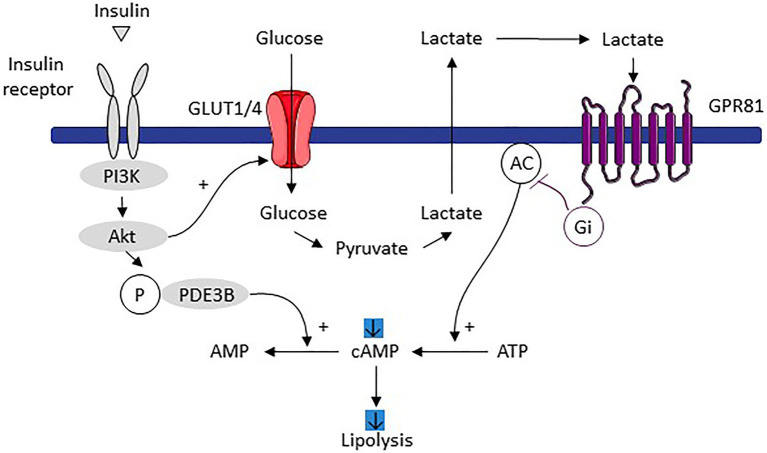
Mechanism of lipolysis inhibition induced by adipocyte dependent lactate production in response to increased glucose utilization stimulated by insulin. Imported glucose following insulin signaling is converted to lactate and then exported. By an autocrine/paracrine action, lactate activates the GPR81 receptor which, through its effects on adenylate cyclase, decreases the level of cAMP and therefore lipolysis. Adapted from Ahmed et al. (2010). Pl3K, phosphoinositide 3-kinase; POE3B, phosphodiesterase 3B; AMPc, cyclic adenosine monophosphate; ATP, adenosine triphosphate; AC, adenylate cyclase; and GPR81, G protein-coupled receptors associated with inhibitory regulative G-protein (Gi).

White adipocytes convert a significant part of the metabolized glucose into lactate (Hagstrom et al., 1990; DiGirolamo et al., 1992; Krycer et al., 2019). The magnitude of the glucose conversion to lactate can reach 50–70% of total glucose metabolized (DiGirolamo et al., 1992). Several signaling pathways regulate this glycolytic pathway such as the insulin-dependent signaling, the FOXK1/2 transcription factors being recently highlighted as important molecular mediators (Sukonina et al., 2019). In addition to adipocytes, the stromal vascular fraction of white adipose tissues is also an important lactate producer, as demonstrated in cells isolated from several rat adipose tissues (Rotondo et al., 2019). Lactate regulates white adipose cell biology and metabolic activity through paracrine and autocrine effects, by acting as a metabolic substrate feeding lipogenesis as a lipid precursor (Katz and Wals, 1974; Francendese and Digirolamo, 1981; Chen et al., 2016) and by stimulating the adipogenic differentiation program of preadipocytes, as recently demonstrated (Harada et al., 2018). This is consistent with the increase in the MCT expression along white adipocyte differentiation (Petersen et al., 2017). Lactate also inhibits the lipolytic activity of white adipocytes by binding to the Gi-coupled G protein-coupled receptor (GPR81; Ahmed et al., 2010). In brief, lactate effects are linked to those of insulin, one of the main lipolysis inhibitors. Insulin increases glucose utilization, a large part of this glucose is converted into lactate that is exported. In an autocrine/paracrine manner, lactate binds to GPR81 that – through its effects on adenylate cyclase – induces a decrease in the level of cAMP and therefore lipolysis (Ahmed et al., 2010; Figure 3). Lactate might thus be observed as a metabolic signal, promoting white adipogenesis and anabolism, to increase the energy storage capacities of white adipose tissues.

**Figure 4:**
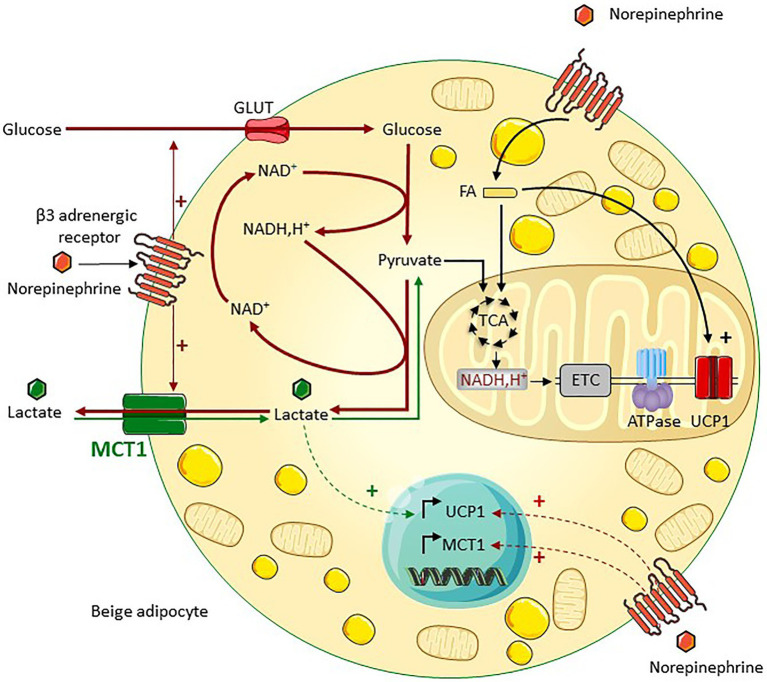
MCT1 is a key regulator of lactate bidirectional fluxes in beige adipocytes. MCT1, which is expressed at the cell surface of beige adipocytes, sustains bidirectional lactate fluxes. Lactate export, which is predominant under noradrenergic stimulation such as cold exposure, supports glycolysis through NAD^+^ regeneration (red arrows). Concomitant lactate import occurs (green arrows), which fuels the oxidative metabolism and promotes the induction of UCPl, thereby increasing the oxidative capacity of beige adipocytes (adapted from Lagarde et al., 2020). ETC, electron transport chain; ATPase, ATP synthase; and FA, fatty acids.

Despite a very different metabolic profile and much greater oxidative capacities, brown adipose tissue is also a major site of lactate production. Although glucose consumption feeds the oxidative metabolism of brown adipose tissue (Wang et al., 2020b), a large amount of this glucose is converted into lactate and exported (Ma and Foster, 1986; Schweizer et al., 2018; Weir et al., 2018), in particular during cold/noradrenergic stimulation. Intense glycolysis ensured by the export of lactate may feed multiple metabolic pathways in brown adipocytes activated by cold, including ATP production (i.e., to compensate for the very low mitochondrial ATP production), the pentose phosphate pathway (i.e., redox balance and lipid synthesis), and glycerol production (i.e., lipogenesis; Hankir and Klingenspor, 2018). Stimulation by the β3 adrenergic pathway also increases lactate release by beige adipocytes in an MCT1-dependent manner, and this lactate release is required for the efficient utilization of glucose by beige adipocytes (Lagarde et al., 2020; Figure 4). This is due to the key role of lactate production and export for redox homeostasis, as discussed earlier. The importance of redox homeostasis for thermogenic adipocytes has been recently highlighted (Nguyen et al., 2020). Further highlighting the importance of MCT1-dependent lactate fluxes in beige adipocyte biology, this transporter is expressed by the subpopulation of adipocytes that turn UCP1 positive on cold exposure, making of it as a marker of inducible beige adipocytes (Lagarde et al., 2020). This finding is very reminiscent of what is occurring during the postnatal development of brown adipose tissue, since only adipocytes expressing MCT1 give rise to UCP1^+^ mature brown adipocytes in Syrian hamster (Okamatsu-Ogura et al., 2018). MCT1 is also strongly expressed in brown adipocytes of adult mice (Iwanaga et al., 2009) and supports optogenetically induced nonshivering thermogenesis (Jeong et al., 2018) through the transport of lactate into mitochondria (Jeong et al., 2018).

Concomitantly to the net lactate outgoing flux and even when glucose is not limiting, a significant import of lactate occurs through MCT1, as shown by ^13^C lactate isotopic tracing experiments. From the thermodynamic point of view, lactate exchange through the plasma membrane is representing the transmembrane equilibration of the respective electrochemical gradients of the transported species (i.e., lactate and protons in this case) in the two compartments through the lactate transporter(s). In other words, the transport is reversible, which means that it does operate in the two directions at the same time with a net flux in one particular direction. Importantly, the imported lactate feeds the oxidative metabolism of beige adipocytes (Lagarde et al., 2020), as also shown for white and brown adipose tissues (Hui et al., 2017, 2020). However, the noradrenergic pathway does not increase lactate uptake in beige adipocytes but fosters lactate release, as a result of high increase in glycolytic flux (Lagarde et al., 2020). Glucose might be then preferred over lactate as a circulating substrate feeding thermogenesis during cold exposure. In response to an increase in systemic lactatemia, the import of lactate may be favored in beige adipocytes. The physiological situations where lactate utilization is increased in beige adipocytes to promote their oxidative metabolism are unknown to our knowledge. In addition to the regulation of the metabolic activity of adipocytes and independently of the β3 adrenergic signaling, intracellular lactate regulates the expression of UCP1 by acting on the intracellular redox balance, thereby contributing to the beiging of adipose tissues.

### Lactate as a Signaling Molecule Inducing Beiging

**Figure 5:**
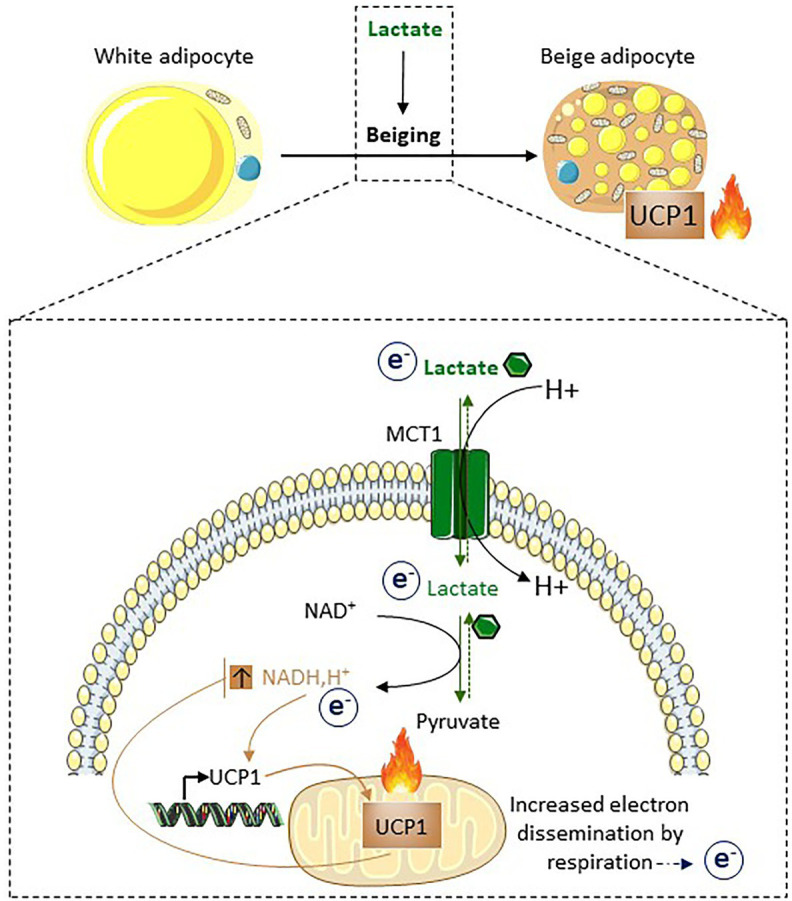
Lactate induces beiging as a way to dissipate redox pressure. Following its import through MCT1, lactate is converted into pyruvate. This conversion is associated with the reduction of NAD^+^ into NADH,H^+^. The increased NADH, H^+^/NAD^+^ ratio triggers UCP1 expression. Due to the properties of UCP1 and its effects on the respiratory chain, UCP1-dependent uncoupling accelerates oxidation of NADH,H^+^ into NAD^+^. Thus, UCP1-dependent uncoupling, in addition to its involvement in non-shivering thermogenesis, may also play an active role in redox homeostasis. Of note, MCT1 is expressed at the cell surface of the subpopulation of adipocytes that will express UCP1 after cold exposure, highlighting it as a marker of inducible beige adipocytes (Carriere et al., 2014; Jeanson et al., 2015; Lagarde et al., 2020).

**Figure 6:**
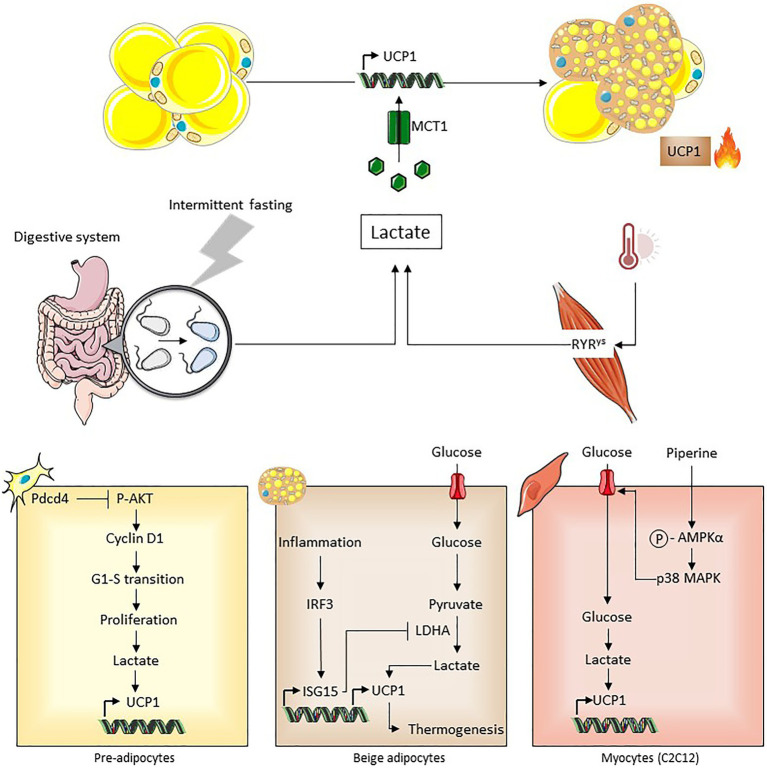
Different contexts associated with lactate-induced beiging. *In vivo*, reprogramming of gut microbiota following intermittent fasting is associated with increased lactate production, which may contribute to beiging that improved systemic metabolic parameters (Li et al., 2017). Increased muscle lactate production caused by a calcium channel mutation is associated with beiging (Wang et al., 2020a). Intracellular production of lactate induced by pdcd4 and piperine in preadipocytes and C2C12 cells respectively, upregulates UCPl expression (Bai et al., 2016; Kim et al., 2017). IRF3/ISG15-mediated inhibition of lactate dehydrogenase decreased lactate-induced UCPl expression thus impairing thermogenesis during inflammation (Yan et al., 2021).

In addition to its role as an energy substrate supporting the oxidative metabolism of beige and brown adipocytes, lactate acts as a signaling molecule triggering their development (Carriere et al., 2014; Figure 5). This effect is independent on the lactate receptor GPR81 but requires redox modifications due to the transport of lactate by MCT1 and increased NADH,H^+^/NAD^+^ ratio subsequent to the lactate conversion into pyruvate by lactate dehydrogenase. Notably, lactate–UCP1 signaling does not occur in the presence of an uncoupling agent (i.e., a state equivalent to activated UCP1), very consistently with the role of NADH,H^+^/NAD^+^ ratio as a metabolic sensor regulating UCP1 expression. Since UCP1-dependent uncoupling accelerates the activity of the respiratory chain by therefore facilitating NADH,H^+^ oxidation and the overall electron flow, we proposed that UCP1-dependent uncoupling may be key for redox stress management (Carriere et al., 2014; Jeanson et al., 2015; Figure 5). This is supported by findings demonstrating that the uncoupling activity of UCP1 is activated by mitochondrial ROS (Chouchani et al., 2016) and that UCP1 controls ROS in brown adipose tissue (Jastroch, 2017). Lactate-induced UCP1 expression could represent an adaptive mechanism where adipocytes acquire increased oxidative capacities to maintain redox homeostasis. The role of lactate as a beiging inducer has been shown in different contexts (Figure 6). The implication of intracellular lactate production in UCP1 induction has been demonstrated in preadipocytes (Bai et al., 2016) and in muscle cells (Kim et al., 2017). It has also been demonstrated that during intermittent fasting, the reprogramming of the intestinal microbiota induces lactate production that contributes to the beiging of white adipose tissue, which plays an important role in the improvement of metabolic profiles (Li et al., 2017). The alteration of lactate-UCP1 signaling has physiopathological consequences, as the inhibition of lactate production through interferon regulatory factor-3 (IRF3)-mediated inhibition of lactate dehydrogenase contributes to the downregulation of UCP1 expression and thermogenesis, in an inflammatory context (Yan et al., 2021). Recently, in a mouse model exhibiting a mutation in the skeletal muscle Ca^2+^ release channel (i.e., RYR1) that induces malignant hyperthermia, an increased production of lactate in muscle is involved in the beiging of white adipose tissue and the activation of brown adipose tissue in an MCT1-dependent manner, which would actively contribute to the hyperthermic phenotype (Wang et al., 2020a). Together, these recent studies highlight the physiological and pathological importance of lactate-induced beiging. In addition, to promote beiging, lactate also induces the expression and secretion of the fibroblast growth factor-21 in both adipocytes (Jeanson et al., 2016) and muscle cells (Villarroya et al., 2018), which is an important regulator of glucose homeostasis and which triggers adaptive responses to reduce metabolic stresses. Thus, lactate, by various independent mechanisms, increases the oxidative capacities of beige adipocytes and stimulates the release of factors enabling adaptation to redox pressure and metabolic stresses.

All these findings highlight how lactate metabolism plays a role in the biology, metabolism, and plasticity of adipose tissues, by acting as a redox stress signal enabling cell adaptation. Interestingly, several studies of the literature show that adipose tissues can regulate systemic lactate metabolism, opening physiopathological perspectives.

### Contribution of Adipose Tissues to the Regulation of Systemic Lactate Metabolism

Several tissues contribute to the regulation of lactate systemic levels, such as muscles, liver, red blood cells, brain, and heart, the rate of lactate release and uptake depending on the physiopathological situations for each tissue (Adeva-Andany et al., 2014). It is known that white adipose tissues are an important site of lactate production that can impact lactate circulating levels (DiGirolamo et al., 1992), probably due, at least in part, to the fact that they represent an important percentage of the total body weight. The quantitative contribution of all adipose depots to total systemic lactate metabolism is, however, unclear and not resolved to our knowledge. Due to the insulin-dependent stimulation of glucose uptake, lactate release by white adipose tissue increases in a postabsorptive state and is likely a major contributor to the increased circulating levels of lactate following insulin or glucose challenge in healthy humans (Hagstrom et al., 1990; Jansson et al., 1994; Qvisth et al., 2007). Lactate release by adipose tissues is further enhanced in elderly and obese subjects (Jansson et al., 1994; Faintrenie and Geloen, 1997) due to their high-fat mass and the decrease in their oxidative capacities, notably due to hypoxia (Trayhurn and Alomar, 2015). Fasting plasma lactate concentration is increased in obese subjects compared with lean control patients, and also in diabetic patients (Lovejoy et al., 1992; Adeva-Andany et al., 2014), which might be an early change in the time course of the disease (Wu et al., 2016b). These results are in agreement with the positive correlation observed between the size of adipocytes and the quantity of lactate produced (DiGirolamo et al., 1992). In the context of obesity, hypertrophic adipocytes with extremely limited oxidative potential and saturated storage capacities are less able to use glucose as a carbon source for oxidative metabolism. Glucose is then massively converted into lactate and exported. Although the role of lactate in the development of insulin resistance needs to be further investigated, lactate could compromise insulin signaling and glucose transport in muscle (Choi et al., 2002). These results suggest that in the context of obesity or aging, the alteration of the oxidative potential of white adipose tissues may increase lactate production by adipocytes, which might participate in the development of metabolic diseases. Using MCT1 heterozygous mice that are resistant to obesity (Lengacher et al., 2013), the transport of lactate into hepatocytes has been shown to contribute to the development of hepatic steatosis (Carneiro et al., 2017), suggesting that during obesity, dysregulated lactate metabolism may participate to the development of metabolic disorders. Among the underlying mechanisms, the acetylation of lactate dehydrogenase B in the liver of high-fat-diet-exposed mice would contribute to impaired lactate clearance and liver steatosis (Wang et al., 2021). Notably, the decrease of beiging in the obesity-associated lactate-rich environment could be due to several mechanisms such as the loss of cellular plasticity and/or the alteration of lactate-sensing mechanisms and impaired lactate fluxes in adipose cells in an obesity context. Although genetic models are definitively required to better investigate the role of adipose tissues in the regulation of lactate systemic lactate fluxes, a transgenic Drosophila model harboring the inhibition of lactate dehydrogenase specifically in the fat body has been recently generated. Very interestingly, these flies exhibited a decrease in circulating levels of lactate, which was associated with an improved body-wide glucose utilization (Krycer et al., 2019), further highlighting the role of adipose tissues in whole-body energy homeostasis through their impact on the circulating levels of lactate.

The role of brown and beige adipose tissues on the regulation of systemic lactate metabolism is poorly described. However, physical exercise that is associated with increased lactate circulating levels induces beiging (Bostrom et al., 2012; Dewal and Stanford, 2019) and increases the expression of MCT1 in brown and beige adipose tissues (De Matteis et al., 2013). This could therefore increase the rate of lactate utilization in these tissues, as a way to manage redox pressure and related oxidative stress. Whether beige and brown adipocytes could act as lactate metabolic sinks as recently proposed for succinate (Mills et al., 2018, 2021) or branched-chain amino acids (Yoneshiro et al., 2019) remain to be demonstrated. This hypothesis may be supported by the positive correlation between plasma lactate and body temperature in five healthy volunteers subjected to physical exercise (Son’kin et al., 2014). Interestingly, the rise in blood lactate levels precedes the rise in temperature. In this study, the highest body and local temperatures at the nape of the neck (i.e., the location of brown adipose tissue in humans) as well as the highest blood lactate levels were observed not during exercise but within minutes afterward. We could envision that during exercise, some of the lactate produced is consumed by the muscle and heart, but that when exercise is stopped, the activation of brown adipose tissue could limit the rise in blood lactate levels.

### Conclusion and Perspectives

Neglected during many years and even considered as a metabolic waste, the multiple roles of lactate as metabolic substrate, redox shuttle, and signaling molecule participate to its rehabilitation as a hub for multiple metabolic pathways notably in adipose tissues. At the whole-body level, the capacities to maintain redox homeostasis on challenges are key for energetic homeostasis. Given their important mass and their high plasticity, adipose tissues appear to be major players in the redox balance and are perfectly adapted to respond to acute and chronic redox stresses. Adipose tissues may act as a redox buffering system by assimilating or disseminating electrons depending on the situation and regulating systemic redox homeostasis through interorgan metabolic dialogs. Although most cells of the organism are capable of transporting lactate, the significant capacities of adipocytes to store energy in excess (i.e., white adipocytes) or to dissipate it in the form of heat (i.e., beige and brown adipocytes) and their high cellular and metabolic plasticity make them cells that are specially adapted to play this redox buffering function. Lactate-dependent cellular responses may be part of the stress-responsive mechanisms enabling adipose cells to acquire an appropriate phenotype to face stresses. Any lack of appropriate response to lactate challenge could have tissue and whole-body repercussions. It is known that in a context of obesity or aging, due to mitochondrial dysfunctions, a decrease in adrenergic tone and modification of inflammatory state, plasticity of adipose tissues, their storage capacity, and their oxidative capacities are reduced. In these pathological contexts, adipose tissues may no longer allow the body to face redox stress and could even contribute to it by increasing lactate release. Investigating the existence of new interorgan communication, which is mediated by lactate or additional metabolites involved in redox homeostasis, and their role during aging or metabolic diseases is definitively an exciting area for future studies.

## Author Contributions

DL designed all the figures. AC supervised and finalized the manuscript. DL, YJ, J-CP, AG, IA, LC, and AC contributed to the design and writing of the manuscript, proofread, and gave comments and suggestions. All authors contributed to the article and approved the submitted version.

### Conflict of Interest

The authors declare that the research was conducted in the absence of any commercial or financial relationships that could be construed as a potential conflict of interest.

## References

[ref1] Adeva-Andany M. Lopez-Ojen M. Funcasta-Calderon R. Ameneiros-Rodriguez E. Donapetry-Garcia C. Vila-Altesor M.. (2014). Comprehensive review on lactate metabolism in human health. Mitochondrion 17, 76–100. 10.1016/j.mito.2014.05.007, PMID: 24929216

[ref2] Ahmed K. Tunaru S. Tang C. Muller M. Gille A. Sassmann A.. (2010). An autocrine lactate loop mediates insulin-dependent inhibition of lipolysis through GPR81. Cell Metab. 11, 311–319. 10.1016/j.cmet.2010.02.012, PMID: 20374963

[ref3] Bai Y. Shang Q. Zhao H. Pan Z. Guo C. Zhang L.. (2016). Pdcd4 restrains the self-renewal and white-to-beige transdifferentiation of adipose-derived stem cells. Cell Death Dis. 7:e2169. 10.1038/cddis.2016.75, PMID: 27031966 PMC4823969

[ref4] Barbatelli G. Murano I. Madsen L. Hao Q. Jimenez M. Kristiansen K.. (2010). The emergence of cold-induced brown adipocytes in mouse white fat depots is determined predominantly by white to brown adipocyte transdifferentiation. Am. J. Physiol. Endocrinol. Metab. 298, E1244–E1253. 10.1152/ajpendo.00600.2009, PMID: 20354155

[ref5] Barreau C. Labit E. Guissard C. Rouquette J. Boizeau M. L. Gani Koumassi S.. (2016). Regionalization of browning revealed by whole subcutaneous adipose tissue imaging. Obesity 24, 1081–1089. 10.1002/oby.21455, PMID: 26999447

[ref6] Bertholet A. M. Kirichok Y. (2017). UCP1: A transporter for H^+^ and fatty acid anions. Biochimie 134, 28–34. 10.1016/j.biochi.2016.10.013, PMID: 27984203 PMC5461058

[ref7] Betz M. J. Enerback S. (2018). Targeting thermogenesis in brown fat and muscle to treat obesity and metabolic disease. Nat. Rev. Endocrinol. 14, 77–87. 10.1038/nrendo.2017.132, PMID: 29052591

[ref8] Bostrom P. Wu J. Jedrychowski M. P. Korde A. Ye L. Lo J. C.. (2012). A PGC1-alpha-dependent myokine that drives brown-fat-like development of white fat and thermogenesis. Nature 481, 463–468. 10.1038/nature10777, PMID: 22237023 PMC3522098

[ref9] Brooks G. A. (2018). The science and translation of lactate shuttle theory. Cell Metab. 27, 757–785. 10.1016/j.cmet.2018.03.008, PMID: 29617642

[ref10] Cannon B. Nedergaard J. (2004). Brown adipose tissue: function and physiological significance. Physiol. Rev. 84, 277–359. 10.1152/physrev.00015.2003, PMID: 14715917

[ref11] Carneiro L. Asrih M. Repond C. Sempoux C. Stehle J. C. Leloup C.. (2017). AMPK activation caused by reduced liver lactate metabolism protects against hepatic steatosis in MCT1 haploinsufficient mice. Mol. Metab. 6, 1625–1633. 10.1016/j.molmet.2017.10.005, PMID: 29092796 PMC5699913

[ref12] Carriere A. Jeanson Y. Berger-Muller S. Andre M. Chenouard V. Arnaud E.. (2014). Browning of white adipose cells by intermediate metabolites: an adaptive mechanism to alleviate redox pressure. Diabetes 63, 3253–3265. 10.2337/db13-1885, PMID: 24789919

[ref13] Carriere A. Lagarde D. Jeanson Y. Portais J. C. Galinier A. Ader I.. (2020). The emerging roles of lactate as a redox substrate and signaling molecule in adipose tissues. J. Physiol. Biochem. 76, 241–250. 10.1007/s13105-019-00723-2, PMID: 31898016

[ref14] Castillo-Armengol J. Fajas L. Lopez-Mejia I. C. (2019). Inter-organ communication: a gatekeeper for metabolic health. EMBO Rep. 20:e47903. 10.15252/embr.201947903, PMID: 31423716 PMC6726901

[ref15] Chen Y. J. Mahieu N. G. Huang X. Singh M. Crawford P. A. Johnson S. L.. (2016). Lactate metabolism is associated with mammalian mitochondria. Nat. Chem. Biol. 12, 937–943. 10.1038/nchembio.2172, PMID: 27618187 PMC5069139

[ref16] Choi C. S. Kim Y. B. Lee F. N. Zabolotny J. M. Kahn B. B. Youn J. H. (2002). Lactate induces insulin resistance in skeletal muscle by suppressing glycolysis and impairing insulin signaling. Am. J. Physiol. Endocrinol. Metab. 283, E233–E240. 10.1152/ajpendo.00557.2001, PMID: 12110527

[ref17] Chouchani E. T. Kajimura S. (2019). Metabolic adaptation and maladaptation in adipose tissue. Nat. Metab. 1, 189–200. 10.1038/s42255-018-0021-8, PMID: 31903450 PMC6941795

[ref18] Chouchani E. T. Kazak L. Jedrychowski M. P. Lu G. Z. Erickson B. K. Szpyt J.. (2016). Mitochondrial ROS regulate thermogenic energy expenditure and sulfenylation of UCP1. Nature 532, 112–116. 10.1038/nature17399, PMID: 27027295 PMC5549630

[ref19] Cinti S. (2012). The adipose organ at a glance. Dis. Model. Mech. 5, 588–594. 10.1242/dmm.009662, PMID: 22915020 PMC3424455

[ref20] Cori C. F. (1981). The glucose-lactic acid cycle and gluconeogenesis. Curr. Top. Cell. Regul. 18, 377–387. 7273846

[ref21] Cori C. F. Cori G. T. (1929). Glycogen formation in the liver from d- and l-lactic acid. J. Biol. Chem. 81, 389–403. 10.1016/S0021-9258(18)83822-4

[ref22] Cousin B. Cinti S. Morroni M. Raimbault S. Ricquier D. Penicaud L.. (1992). Occurrence of brown adipocytes in rat white adipose tissue: molecular and morphological characterization. J. Cell Sci. 103, 931–942. 10.1242/jcs.103.4.931, PMID: 1362571

[ref23] Cypess A. M. Lehman S. Williams G. Tal I. Rodman D. Goldfine A. B.. (2009). Identification and importance of brown adipose tissue in adult humans. N. Engl. J. Med. 360, 1509–1517. 10.1056/NEJMoa0810780, PMID: 19357406 PMC2859951

[ref24] De Matteis R. Lucertini F. Guescini M. Polidori E. Zeppa S. Stocchi V.. (2013). Exercise as a new physiological stimulus for brown adipose tissue activity. Nutr. Metab. Cardiovasc. Dis. 23, 582–590. 10.1016/j.numecd.2012.01.013, PMID: 22633794

[ref25] Dewal R. S. Stanford K. I. (2019). Effects of exercise on brown and beige adipocytes. Biochim. Biophys. Acta Mol. Cell Biol. Lipids 1864, 71–78. 10.1016/j.bbalip.2018.04.013, PMID: 29684558 PMC6292667

[ref26] Dichamp J. Barreau C. Guissard C. Carriere A. Martinez Y. Descombes X.. (2019). 3D analysis of the whole subcutaneous adipose tissue reveals a complex spatial network of interconnected lobules with heterogeneous browning ability. Sci. Rep. 9:6684. 10.1038/s41598-019-43130-9, PMID: 31040317 PMC6491608

[ref27] DiGirolamo M. Newby F. D. Lovejoy J. (1992). Lactate production in adipose tissue: a regulated function with extra-adipose implications. FASEB J. 6, 2405–2412. 10.1096/fasebj.6.7.1563593, PMID: 1563593

[ref28] Dovmark T. H. Saccomano M. Hulikova A. Alves F. Swietach P. (2017). Connexin-43 channels are a pathway for discharging lactate from glycolytic pancreatic ductal adenocarcinoma cells. Oncogene 36, 4538–4550. 10.1038/onc.2017.71, PMID: 28368405 PMC5507299

[ref29] Faintrenie G. Geloen A. (1997). Effect of aging on norepinephrine and phenylephrine stimulated lactate production by white adipocytes. Obes. Res. 5, 100–104. 10.1002/j.1550-8528.1997.tb00649.x, PMID: 9112244

[ref30] Faubert B. Li K. Y. Cai L. Hensley C. T. Kim J. Zacharias L. G.. (2017). Lactate metabolism in human lung tumors. Cell 171, 358–371.e9. 10.1016/j.cell.2017.09.019, PMID: 28985563 PMC5684706

[ref31] Ferguson B. S. Rogatzki M. J. Goodwin M. L. Kane D. A. Rightmire Z. Gladden L. B. (2018). Lactate metabolism: historical context, prior misinterpretations, and current understanding. Eur. J. Appl. Physiol. 118, 691–728. 10.1007/s00421-017-3795-6, PMID: 29322250

[ref32] Francendese A. A. Digirolamo M. (1981). Alternative substrates for triacylglycerol synthesis in isolated adipocytes of different size from the rat. Biochem. J. 194, 377–384. 10.1042/bj1940377, PMID: 7030317 PMC1162760

[ref33] Galinier A. Carriere A. Fernandez Y. Carpene C. Andre M. Caspar-Bauguil S.. (2006). Adipose tissue proadipogenic redox changes in obesity. J. Biol. Chem. 281, 12682–12687. 10.1074/jbc.M506949200, PMID: 16377639

[ref34] Gaude E. Schmidt C. Gammage P. A. Dugourd A. Blacker T. Chew S. P.. (2018). NADH shuttling couples cytosolic reductive carboxylation of glutamine with glycolysis in cells with mitochondrial dysfunction. Mol. Cell 69, 581–593.e7. 10.1016/j.molcel.2018.01.034, PMID: 29452638 PMC5823973

[ref35] Hagstrom E. Arner P. Ungerstedt U. Bolinder J. (1990). Subcutaneous adipose tissue: a source of lactate production after glucose ingestion in humans. Am. J. Phys. 258, E888–E893. 10.1152/ajpendo.1990.258.5.E888, PMID: 2333992

[ref36] Halestrap A. P. (2013). The SLC16 gene family—structure, role and regulation in health and disease. Mol. Asp. Med. 34, 337–349. 10.1016/j.mam.2012.05.003, PMID: 23506875

[ref37] Han J. Meng Q. Shen L. Wu G. (2018). Interleukin-6 induces fat loss in cancer cachexia by promoting white adipose tissue lipolysis and browning. Lipids Health Dis. 17:14. 10.1186/s12944-018-0657-0, PMID: 29338749 PMC5771021

[ref38] Hankir M. K. Klingenspor M. (2018). Brown adipocyte glucose metabolism: a heated subject. EMBO Rep. 19:e46404. 10.15252/embr.201846404, PMID: 30135070 PMC6123662

[ref39] Harada N. Hirano I. Inui H. Yamaji R. (2018). Stereoselective effects of lactate enantiomers on the enhancement of 3T3-L1 adipocyte differentiation. Biochem. Biophys. Res. Commun. 498, 105–110. 10.1016/j.bbrc.2018.02.198, PMID: 29501496

[ref40] Hashimoto T. Hussien R. Oommen S. Gohil K. Brooks G. A. (2007). Lactate sensitive transcription factor network in L6 cells: activation of MCT1 and mitochondrial biogenesis. FASEB J. 21, 2602–2612. 10.1096/fj.07-8174com, PMID: 17395833

[ref41] Holmstrom K. M. Finkel T. (2014). Cellular mechanisms and physiological consequences of redox-dependent signalling. Nat. Rev. Mol. Cell Biol. 15, 411–421. 10.1038/nrm3801, PMID: 24854789

[ref42] Hosios A. M. Vander Heiden M. G. (2018). The redox requirements of proliferating mammalian cells. J. Biol. Chem. 293, 7490–7498. 10.1074/jbc.TM117.000239, PMID: 29339555 PMC5961062

[ref43] Hui S. Cowan A. J. Zeng X. Yang L. TeSlaa T. Li X.. (2020). Quantitative fluxomics of circulating metabolites. Cell Metab. 32, 676–688.e4. 10.1016/j.cmet.2020.07.013, PMID: 32791100 PMC7544659

[ref44] Hui S. Ghergurovich J. M. Morscher R. J. Jang C. Teng X. Lu W.. (2017). Glucose feeds the TCA cycle via circulating lactate. Nature 551, 115–118. 10.1038/nature24057, PMID: 29045397 PMC5898814

[ref45] Hussain M. F. Roesler A. Kazak L. (2020). Regulation of adipocyte thermogenesis: mechanisms controlling obesity. FEBS J. 287, 3370–3385. 10.1111/febs.15331, PMID: 32301220

[ref46] Iwanaga T. Kuchiiwa T. Saito M. (2009). Histochemical demonstration of monocarboxylate transporters in mouse brown adipose tissue. Biomed. Res. 30, 217–225. 10.2220/biomedres.30.217, PMID: 19729852

[ref47] Jansson P. A. Larsson A. Smith U. Lonnroth P. (1994). Lactate release from the subcutaneous tissue in lean and obese men. J. Clin. Invest. 93, 240–246. 10.1172/JCI116951, PMID: 8282793 PMC293758

[ref48] Jastroch M. (2017). Uncoupling protein 1 controls reactive oxygen species in brown adipose tissue. Proc. Natl. Acad. Sci. U. S. A. 114, 7744–7746. 10.1073/pnas.1709064114, PMID: 28710335 PMC5544340

[ref49] Jeanson Y. Carriere A. Casteilla L. (2015). A new role for browning as a redox and stress adaptive mechanism? Front. Endocrinol. 6:158. 10.3389/fendo.2015.00158, PMID: 26500607 PMC4598589

[ref50] Jeanson Y. Ribas F. Galinier A. Arnaud E. Ducos M. Andre M.. (2016). Lactate induces FGF21 expression in adipocytes through a p38-MAPK pathway. Biochem. J. 473, 685–692. 10.1042/BJ20150808, PMID: 26769382

[ref51] Jeong J. H. Chang J. S. Jo Y. H. (2018). Intracellular glycolysis in brown adipose tissue is essential for optogenetically induced nonshivering thermogenesis in mice. Sci. Rep. 8:6672. 10.1038/s41598-018-25265-3, PMID: 29704006 PMC5923201

[ref52] Katz J. Wals P. A. (1974). Lipogenesis from lactate in rat adipose tissue. Biochim. Biophys. Acta 348, 344–356. 10.1016/0005-2760(74)90214-8, PMID: 4847561

[ref53] Kim N. Nam M. Kang M. S. Lee J. O. Lee Y. W. Hwang G. S.. (2017). Piperine regulates UCP1 through the AMPK pathway by generating intracellular lactate production in muscle cells. Sci. Rep. 7:41066. 10.1038/srep41066, PMID: 28117414 PMC5259784

[ref54] Kir S. White J. P. Kleiner S. Kazak L. Cohen P. Baracos V. E.. (2014). Tumour-derived PTH-related protein triggers adipose tissue browning and cancer cachexia. Nature 513, 100–104. 10.1038/nature13528, PMID: 25043053 PMC4224962

[ref55] Krycer J. R. Quek L. E. Francis D. Fazakerley D. J. Elkington S. D. Diaz-Vegas A.. (2019). Lactate production is a prioritised feature of adipocyte metabolism. J. Biol. Chem. 295, 83–98. 10.1074/jbc.RA119.011178, PMID: 31690627 PMC6952601

[ref56] Lafontan M. Langin D. (2009). Lipolysis and lipid mobilization in human adipose tissue. Prog. Lipid Res. 48, 275–297. 10.1016/j.plipres.2009.05.001, PMID: 19464318

[ref57] Lagarde D. Jeanson Y. Barreau C. Moro C. Peyriga L. Cahoreau E.. (2020). Lactate fluxes mediated by the monocarboxylate transporter-1 are key determinants of the metabolic activity of beige adipocytes. J. Biol. Chem. 296:100137. 10.1074/jbc.RA120.016303, PMID: 33268383 PMC7949083

[ref58] Lee Y. H. Petkova A. P. Mottillo E. P. Granneman J. G. (2012). In vivo identification of bipotential adipocyte progenitors recruited by beta3-adrenoceptor activation and high-fat feeding. Cell Metab. 15, 480–491. 10.1016/j.cmet.2012.03.009, PMID: 22482730 PMC3322390

[ref59] Lengacher S. Nehiri-Sitayeb T. Steiner N. Carneiro L. Favrod C. Preitner F.. (2013). Resistance to diet-induced obesity and associated metabolic perturbations in haploinsufficient monocarboxylate transporter 1 mice. PLoS One 8:e82505. 10.1371/journal.pone.0082505, PMID: 24367518 PMC3867350

[ref60] Leveillard T. Philp N. J. Sennlaub F. (2019). Is retinal metabolic dysfunction at the center of the pathogenesis of age-related macular degeneration? Int. J. Mol. Sci. 20:762. 10.3390/ijms20030762, PMID: 30754662 PMC6387069

[ref61] Li G. Xie C. Lu S. Nichols R. G. Tian Y. Li L.. (2017). Intermittent fasting promotes white adipose browning and decreases obesity by shaping the gut microbiota. Cell Metab. 26, 672–685.e4. 10.1016/j.cmet.2017.08.019, PMID: 28918936 PMC5668683

[ref62] Liu K. Lin L. Li Q. Xue Y. Zheng F. Wang G.. (2020). Scd1 controls de novo beige fat biogenesis through succinate-dependent regulation of mitochondrial complex II. Proc. Natl. Acad. Sci. U. S. A. 117, 2462–2472. 10.1073/pnas.1914553117, PMID: 31953260 PMC7007576

[ref63] Loncar D. (1991). Convertible adipose tissue in mice. Cell Tissue Res. 266, 149–161. 10.1007/BF00678721, PMID: 1747909

[ref64] Lovejoy J. Newby F. D. Gebhart S. S. DiGirolamo M. (1992). Insulin resistance in obesity is associated with elevated basal lactate levels and diminished lactate appearance following intravenous glucose and insulin. Metab. Clin. Exp. 41, 22–27. 10.1016/0026-0495(92)90185-D 1538640

[ref65] Ma S. W. Foster D. O. (1986). Uptake of glucose and release of fatty acids and glycerol by rat brown adipose tissue in vivo. Can. J. Physiol. Pharmacol. 64, 609–614. 10.1139/y86-101, PMID: 3730946

[ref66] Mills E. L. Harmon C. Jedrychowski M. P. Xiao H. Garrity R. Tran N. V.. (2021). UCP1 governs liver extracellular succinate and inflammatory pathogenesis. Nat. Metab. 3, 604–617. 10.1038/s42255-021-00389-5, PMID: 34002097 PMC8207988

[ref67] Mills E. L. Pierce K. A. Jedrychowski M. P. Garrity R. Winther S. Vidoni S.. (2018). Accumulation of succinate controls activation of adipose tissue thermogenesis. Nature 560, 102–106. 10.1038/s41586-018-0353-2, PMID: 30022159 PMC7045287

[ref68] Nguyen H. P. Yi D. Lin F. Viscarra J. A. Tabuchi C. Ngo K.. (2020). Aifm2, a NADH oxidase supports robust glycolysis and is required for cold- and diet-induced thermogenesis. Mol. Cell 77, 600–617.e4. 10.1016/j.molcel.2019.12.002, PMID: 31952989 PMC7031813

[ref69] Nicholls D. G. Locke R. M. (1984). Thermogenic mechanisms in brown fat. Physiol. Rev. 64, 1–64. 10.1152/physrev.1984.64.1.1, PMID: 6320232

[ref70] Okamatsu-Ogura Y. Nio-Kobayashi J. Nagaya K. Tsubota A. Kimura K. (2018). Brown adipocytes postnatally arise through both differentiation from progenitors and conversion from white adipocytes in Syrian hamster. J. Appl. Physiol. 124, 99–108. 10.1152/japplphysiol.00622.2017, PMID: 28982944

[ref71] Park J. Shin S. Liu L. Jahan I. Ong S. G. Xu P.. (2021). Progenitor-like characteristics in a subgroup of UCP1+ cells within white adipose tissue. Dev. Cell 56, 985–999.e4. 10.1016/j.devcel.2021.02.018, PMID: 33711247 PMC8026751

[ref72] Pellerin L. Magistretti P. J. (2012). Sweet sixteen for ANLS. J. Cereb. Blood Flow Metab. 32, 1152–1166. 10.1038/jcbfm.2011.149, PMID: 22027938 PMC3390819

[ref73] Perez-Escuredo J. Van Hee V. F. Sboarina M. Falces J. Payen V. L. Pellerin L.. (2016). Monocarboxylate transporters in the brain and in cancer. Biochim. Biophys. Acta 1863, 2481–2497. 10.1016/j.bbamcr.2016.03.013, PMID: 26993058 PMC4990061

[ref505] Petersen C. Nielsen M. D. Andersen E. S. Basse A. L. Isidor M. S. Markussen L. K.. (2017). MCT1 and MCT4 Expression and Lactate Flux Activity Increase During White and Brown Adipogenesis and Impact Adipocyte Metabolism. Sci. Rep. 7:13101. 10.1038/s41598-017-13298-z, PMID: 29026134 PMC5638914

[ref74] Petruzzelli M. Schweiger M. Schreiber R. Campos-Olivas R. Tsoli M. Allen J.. (2014). A switch from white to brown fat increases energy expenditure in cancer-associated cachexia. Cell Metab. 20, 433–447. 10.1016/j.cmet.2014.06.011, PMID: 25043816

[ref75] Porter C. Herndon D. N. Bhattarai N. Ogunbileje J. O. Szczesny B. Szabo C.. (2015). Severe burn injury induces thermogenically functional mitochondria in murine white adipose tissue. Shock 44, 258–264. 10.1097/SHK.0000000000000410, PMID: 26009824 PMC4537662

[ref76] Pucino V. Certo M. Bulusu V. Cucchi D. Goldmann K. Pontarini E.. (2019). Lactate buildup at the site of chronic inflammation promotes disease by inducing CD4^+^ T cell metabolic rewiring. Cell Metab. 30, 1055–1074.e8. 10.1016/j.cmet.2019.10.004, PMID: 31708446 PMC6899510

[ref77] Qvisth V. Hagstrom-Toft E. Moberg E. Sjoberg S. Bolinder J. (2007). Lactate release from adipose tissue and skeletal muscle in vivo: defective insulin regulation in insulin-resistant obese women. Am. J. Physiol. Endocrinol. Metab. 292, E709–E714. 10.1152/ajpendo.00104.2006, PMID: 17077346

[ref78] Rabinowitz J. D. Enerback S. (2020). Lactate: the ugly duckling of energy metabolism. Nat. Metab. 2, 566–571. 10.1038/s42255-020-0243-4, PMID: 32694798 PMC7983055

[ref79] Rodriguez-Colman M. J. Schewe M. Meerlo M. Stigter E. Gerrits J. Pras-Raves M.. (2017). Interplay between metabolic identities in the intestinal crypt supports stem cell function. Nature 543, 424–427. 10.1038/nature21673, PMID: 28273069

[ref80] Roesler A. Kazak L. (2020). UCP1-independent thermogenesis. Biochem. J. 477, 709–725. 10.1042/BCJ20190463, PMID: 32059055

[ref81] Rosenwald M. Perdikari A. Rulicke T. Wolfrum C. (2013). Bi-directional interconversion of brite and white adipocytes. Nat. Cell Biol. 15, 659–667. 10.1038/ncb2740, PMID: 23624403

[ref82] Rotondo F. Ho-Palma A. C. Romero M. D. M. Remesar X. Fernandez-Lopez J. A. Alemany M. (2019). Higher lactate production from glucose in cultured adipose nucleated stromal cells than for rat adipocytes. Adipocytes 8, 61–76. 10.1080/21623945.2019.1569448, PMID: 30676233 PMC6768231

[ref83] Sahuri-Arisoylu M. Brody L. P. Parkinson J. R. Parkes H. Navaratnam N. Miller A. D.. (2016). Reprogramming of hepatic fat accumulation and ‘browning’ of adipose tissue by the short-chain fatty acid acetate. Int. J. Obes. 40, 955–963. 10.1038/ijo.2016.23, PMID: 26975441

[ref84] Schweizer S. Oeckl J. Klingenspor M. Fromme T. (2018). Substrate fluxes in brown adipocytes upon adrenergic stimulation and uncoupling protein 1 ablation. Life Sci. Alliance 1:e201800136. 10.26508/lsa.201800136, PMID: 30456392 PMC6238590

[ref85] Sidossis L. Kajimura S. (2015). Brown and beige fat in humans: thermogenic adipocytes that control energy and glucose homeostasis. J. Clin. Invest. 125, 478–486. 10.1172/JCI78362, PMID: 25642708 PMC4319444

[ref86] Sidossis L. S. Porter C. Saraf M. K. Borsheim E. Radhakrishnan R. S. Chao T.. (2015). Browning of subcutaneous white adipose tissue in humans after severe adrenergic stress. Cell Metab. 22, 219–227. 10.1016/j.cmet.2015.06.022, PMID: 26244931 PMC4541608

[ref87] Son’kin V. D. Akimov E. B. Andreev R. S. Yakushkin A. V. Kozlov A. V. (2014). “Brown adipose tissue participate in lactate utilization during muscular work,” in *Proceedings of the 2nd International Congress on Sports Sciences Research and Technology Support (icSPORTS-2014)*. October 24-26, 2014 (Rome: Italy), 97–102.

[ref88] Sonveaux P. Vegran F. Schroeder T. Wergin M. C. Verrax J. Rabbani Z. N.. (2008). Targeting lactate-fueled respiration selectively kills hypoxic tumor cells in mice. J. Clin. Invest. 118, 3930–3942. 10.1172/JCI36843, PMID: 19033663 PMC2582933

[ref89] Sukonina V. Ma H. Zhang W. Bartesaghi S. Subhash S. Heglind M.. (2019). FOXK1 and FOXK2 regulate aerobic glycolysis. Nature 566, 279–283. 10.1038/s41586-019-0900-5, PMID: 30700909

[ref90] Trayhurn P. Alomar S. Y. (2015). Oxygen deprivation and the cellular response to hypoxia in adipocytes—perspectives on white and brown adipose tissues in obesity. Front. Endocrinol. 6:19. 10.3389/fendo.2015.00019, PMID: 25745415 PMC4333869

[ref91] van Marken Lichtenbelt W. D. Vanhommerig J. W. Smulders N. M. Drossaerts J. M. Kemerink G. J. Bouvy N. D.. (2009). Cold-activated brown adipose tissue in healthy men. N. Engl. J. Med. 360, 1500–1508. 10.1056/NEJMoa0808718, PMID: 19357405

[ref92] Villarroya J. Campderros L. Ribas-Aulinas F. Carriere A. Casteilla L. Giralt M.. (2018). Lactate induces expression and secretion of fibroblast growth factor-21 by muscle cells. Endocrine 61, 165–168. 10.1007/s12020-018-1612-6, PMID: 29704156

[ref93] Virtanen K. A. Lidell M. E. Orava J. Heglind M. Westergren R. Niemi T.. (2009). Functional brown adipose tissue in healthy adults. N. Engl. J. Med. 360, 1518–1525. 10.1056/NEJMoa0808949, PMID: 19357407

[ref94] Wang T. Chen K. Yao W. Zheng R. He Q. Xia J.. (2021). Acetylation of lactate dehydrogenase B drives NAFLD progression by impairing lactate clearance. J. Hepatol. 74, 1038–1052. 10.1016/j.jhep.2020.11.028, PMID: 33248168

[ref95] Wang H. J. Lee C. S. Yee R. S. Z. Groom L. Friedman I. Babcock L.. (2020a). Adaptive thermogenesis enhances the life-threatening response to heat in mice with an Ryr1 mutation. Nat. Commun. 11:5099. 10.1038/s41467-020-18865-z, PMID: 33037202 PMC7547078

[ref96] Wang Z. Ning T. Song A. Rutter J. Wang Q. A. Jiang L. (2020b). Chronic cold exposure enhances glucose oxidation in brown adipose tissue. EMBO Rep. 21:e50085. 10.15252/embr.202050085, PMID: 33043581 PMC7645266

[ref97] Wang Q. A. Tao C. Gupta R. K. Scherer P. E. (2013). Tracking adipogenesis during white adipose tissue development, expansion and regeneration. Nat. Med. 19, 1338–1344. 10.1038/nm.3324, PMID: 23995282 PMC4075943

[ref98] Weir G. Ramage L. E. Akyol M. Rhodes J. K. Kyle C. J. Fletcher A. M.. (2018). Substantial metabolic activity of human brown adipose tissue during warm conditions and cold-induced lipolysis of local triglycerides. Cell Metab. 27, 1348–1355.e4. 10.1016/j.cmet.2018.04.020, PMID: 29805098 PMC5988566

[ref99] Wu Y. Dong Y. Atefi M. Liu Y. Elshimali Y. Vadgama J. V. (2016b). Lactate, a neglected factor for diabetes and cancer interaction. Mediat. Inflamm. 2016:6456018. 10.1155/2016/6456018, PMID: 28077918 PMC5203906

[ref100] Wu J. Jin Z. Zheng H. Yan L. J. (2016a). Sources and implications of NADH/NAD^+^ redox imbalance in diabetes and its complications. Diabetes Metab. Syndr. Obes. 9, 145–153. 10.2147/DMSO.S106087, PMID: 27274295 PMC4869616

[ref101] Xiao W. Wang R. S. Handy D. E. Loscalzo J. (2018). NAD(H) and NADP(H) redox couples and cellular energy metabolism. Antioxid. Redox Signal. 28, 251–272. 10.1089/ars.2017.7216, PMID: 28648096 PMC5737637

[ref102] Yan S. Kumari M. Xiao H. Jacobs C. Kochumon S. Jedrychowski M.. (2021). IRF3 reduces adipose thermogenesis via ISG15-mediated reprogramming of glycolysis. J. Clin. Invest. 131:e144888. 10.1172/JCI144888, PMID: 33571167 PMC8011904

[ref103] Yoneshiro T. Wang Q. Tajima K. Matsushita M. Maki H. Igarashi K.. (2019). BCAA catabolism in brown fat controls energy homeostasis through SLC25A44. Nature 572, 614–619. 10.1038/s41586-019-1503-x, PMID: 31435015 PMC6715529

[ref104] Young P. Arch J. R. Ashwell M. (1984). Brown adipose tissue in the parametrial fat pad of the mouse. FEBS Lett. 167, 10–14. 10.1016/0014-5793(84)80822-4, PMID: 6698197

[ref105] Zhang J. Muri J. Fitzgerald G. Gorski T. Gianni-Barrera R. Masschelein E.. (2020). Endothelial lactate controls muscle regeneration from ischemia by inducing M2-like macrophage polarization. Cell Metab. 31, 1136–1153.e7. 10.1016/j.cmet.2020.05.004, PMID: 32492393 PMC7267778

